# Cultural Differences in Interpersonal Emotion Regulation

**DOI:** 10.3389/fpsyg.2019.00999

**Published:** 2019-05-10

**Authors:** Belinda J. Liddell, Emma N. Williams

**Affiliations:** School of Psychology, University of New South Wales, Sydney, NSW, Australia

**Keywords:** culture, emotion regulation, interpersonal, reappraisal, self-construal, collectivism and individualism, heart rate variability, negative affect

## Abstract

Cultural differences exist in the use of emotion regulation (ER) strategies, but the focus to date has been on intrapersonal ER strategies such as cognitive reappraisal. An emerging literature highlights the importance of interpersonal ER, which utilizes social cues to facilitate the regulation of emotional states. In cultures that place high value on social interconnectedness as integral to their collectivistic self-construal, including East Asian cultures, interpersonal ER strategies may be particularly effective in reducing negative affect but this has not been previously tested. In this study, two groups comprising East Asian (*n* = 48) and Western European (*n* = 38) participants were randomly assigned to receive a priming narration depicting the use of either interpersonal (e.g., social modeling, perspective taking) or intrapersonal (e.g., cognitive reappraisal) ER strategies during a stressful experience. They were then instructed to utilize similar ER strategies in an emotion reactivity task during which they viewed high arousing negative pictorial stimuli while their heart rate (HR), heart rate variability (high frequency power – HF-HRV) and subjective affective states were measured. First we found that the East Asian group reported higher use of interpersonal ER strategies of social modeling and perspective taking in daily life. During the experimental interpersonal prime exposure, the East Asian group showed elevated HF-HRV (relative to baseline) compared to the Western European group, indicating more adaptive ER, but this pattern was not sustained during the reactivity or recovery phases. Instead, the East Asian group demonstrated increased HF-HRV and decreased HR across both prime conditions. The East Asian group also showed greater decreases in positive affect across the course of the experiment. Furthermore, individual differences in social modeling and individualistic self-construal moderated the effect of the ER prime in the East Asian group at trend levels, and main effects for perspective taking and reappraisal were observed in the Western European group. The findings support the notion that engaging in interpersonal ER strategies may be more beneficial for East Asian groups when immediately exposed to a stressful situation, as these strategies are congruent with cultural context and preferences, but our priming methodology may have limited the longer-term benefits.

## Introduction

Emotion regulation (ER) functions to shape how we respond to, express and manage our emotional responses to external and internal events. A multi-faceted process, ER works by increasing, decreasing or maintaining the intensity or experience of an affective state in order to navigate our social environment, cope with internal stress or external threats (Gross and Thompson, 2007; Aldao and Nolen-Hoeksema, 2012; Gross, 2013). Theoretical and empirical research has focused largely on intrapersonal (i.e., within person or internal) ER strategies – such as cognitive reappraisal, distraction or suppression, and their effects on reducing negative affect (McRae, 2016) or the stress response (Troy et al., 2013) by directing attention, facilitating cognitive change or modulating affective responses (Webb et al., 2012). While the central models of ER, such as the Process model, focus on certain strategies such as cognitive reappraisal as being the most effective intrapersonal ER strategies in decreasing negative emotions and promoting coping (Gross and Thompson, 2007; Webb et al., 2012), such strategies may not be universally beneficial (Bonanno and Burton, 2013).

Research suggests that social context is critical to informing the effectiveness of ER strategies (Bonanno and Burton, 2013), and this appears to extend to cultural factors. Cultural psychology has placed the conceptualization of the self (known as “self-construal”) as central to understanding cultural differences in behavior, thought, and emotion (Markus and Kitayama, 2010; De Leersnyder et al., 2013). Western-based cultural groups tend to hold a self-construal that is individualistic, where the self is perceived as independent from others, valuing autonomy, self-advancement, and placing the unique self as the central reference point for guiding behavior and emotions (Gore and Cross, 2010; Markus and Kitayama, 2010). By contrast, non-Western cultural groups, including East Asian (EA) cultures, are more likely to hold a collectivistic self-construal where the self is viewed as interdependent with others. In this context, the self is conceptualized as being highly interconnected to the external social environment, where social responsibility and harmony is a primary motivation for behavior and emotion (Cross et al., 2011). These cultural differences in self-construal influence beliefs in what constitutes a “good person,” and the value placed on the wellbeing of the individual relative to the social group (Gore and Cross, 2010; De Leersnyder et al., 2013).

Research has shown that individuals engage in and benefit from employing intrapersonal ER strategies that are consistent with cultural goals and self-concept (Matsumoto et al., 2008; Ford and Mauss, 2015). As such, studies have demonstrated that the relative benefits of cognitive reappraisal or suppression are influenced by culture (Ford and Mauss, 2015). Cognitive reappraisal appears more effective than suppression in managing negative affect in Western cultural groups. However, in cultures that value inter-connectedness, such as EA groups, there appears to be a preference for emotional suppression as this reduces the risk of disrupting group harmony by mitigating the impact of negative emotional states on others (Butler et al., 2007; Matsumoto et al., 2008; Ford and Mauss, 2015). For example, self-report studies have found positive correlations between use of emotional suppression and depressed mood scores in European American groups, but not in Chinese participants (Soto et al., 2011), whereas another study found that use of suppression was correlated with value placed on interpersonal harmony in a Chinese cohort (Wei et al., 2013). Empirical findings support these observations. For example, one study found that habitual use of suppression was associated with self-protective goals and higher negative affect in European Americans, and induced suppression was associated with poor interpersonal responding and adverse perceptions of others; such patterns were reduced in Asian participants (Butler et al., 2007). Another study found that Asian Americans with a strong preference for emotional control demonstrated a down-regulation pattern of cardiovascular responding during an anger provocation task, whereas European Americans who also valued emotional control, did not demonstrate such a down-regulation response (Mauss and Butler, 2010). These studies strongly suggest that culture is an imperative factor in understanding how individuals experience, express and regulate emotions.

Culture may also influence the use of interpersonal (i.e., external, or person-to-person) ER practices, but thus far, it is unknown whether there are cultural differences in the implementation or benefit derived from engaging in interpersonal ER strategies. More broadly, there has been relatively less attention paid to the interpersonal aspects of ER compared to intrapersonal strategies. The emerging notion of interpersonal ER seeks to incorporate the social factors that influence ER processes (Zaki and Williams, 2013; Reeck et al., 2016). Interpersonal ER refers to the social and interpersonal processes by which an individual’s internal emotional states are regulated by others (Hofmann, 2014; Hofmann et al., 2016). As such, interpersonal ER can take several forms. For instance, intrinsic interpersonal ER strategies could include “labeling” where an individual labels their emotions to help describe their feelings to another person, thus gaining awareness, acknowledgment and assessment of their own internal emotional states. Other examples include social modeling – observing what others do in a similarly challenging situation, or perspective taking – having others assist in reframing or reflecting the emotional situation (Zaki and Williams, 2013; Hofmann et al., 2016; Reeck et al., 2016; Dore et al., 2017). By contrast, extrinsic interpersonal ER processes may involve active attempts to regulate the emotions of others via feedback or prosocial acts (Zaki and Williams, 2013). This study will focus on the intrinsic form of interpersonal ER.

While there has been some theoretical development of these concepts (Zaki and Williams, 2013; Reeck et al., 2016), alongside the formulation of a psychometric measures (Hofmann et al., 2016; Williams et al., 2018), there have only been a small number of studies investigating the effectiveness of and mechanisms underpinning interpersonal ER practices. For instance, Levy-Gigi and Shamay-Tsoory (2017) compared two conditions – one where a partner provided suggestions about how to deal with a stressor or second, where participants simply dealt with a stressor independently without social feedback. This study found that the former interpersonal ER condition resulted in a greater reduction in self-reported psychological distress compared to the independent condition. Another study found that individuals providing reappraisal and acceptance-related feedback to an online community who had shared stressful life experiences (i.e., extrinsic interpersonal ER) showed increased use of reappraisal and decreased depressive symptoms, highlighting the benefit of perspective-taking interpersonal strategies (Dore et al., 2017). A third series of studies found that those who tended to view interpersonal ER practices as helpful, received greater benefit from social support following strong emotional experiences – regardless whether these events were positive or negative (Williams et al., 2018). These studies suggest that there is benefit from receiving social input from others while managing strong emotions, and that interpersonal aspects of ER are important to human self-regulation.

An outstanding empirical question is whether culture also affects how interpersonal ER strategies are engaged. Given a key mechanism differentiating cultural groups in terms of emotional expression, perception and social function is self-construal (Kitayama and Markus, 2000; Tsai et al., 2006; De Leersnyder et al., 2014), which determines how one sees themselves in relation to one’s social world, we hypothesize that such cultural factors could modulate the effectiveness of engaging in interpersonal ER strategies. For example, because collectivistic cultures are more focused on social interdependence with others, these groups may be more sensitive to social cues, and therefore profit from utilizing interpersonal strategies to reduce negative emotional states. There is some emerging evidence to suggest that EA groups are more interpersonally sensitive in terms of ER. For example, EA cultural groups have been found to engage more in social perspective taking (Ma et al., 2014), display stronger signs of empathy for others (Cheon et al., 2011; de Greck et al., 2012), and value implicit over explicit social support (Taylor et al., 2007) relative to Western-based cultural groups. These studies alone suggest culture may be an important factor in moderating the implementation and benefit derived from engaging interpersonal ER strategies, but this notion has not been investigated empirically.

This study aims to examine this question by comparing engagement in interpersonal ER practices in groups of Western European (WE) and EA participants. First, we were interested in whether groups differed in self-report measures of habitual use of intrapersonal and interpersonal ER strategies. In our first hypothesis, we predicted that EA participants would report engaging in interpersonal strategies such as perspective taking and social modeling more frequently than the WE cohort, due to stronger levels of collectivistic self-construal and interdependent social orientation.

Second, we were interested in whether groups differed on the impact of engaging in interpersonal ER strategies to manage affective and physiological reactions to negative cues. Effective ER can be measured via self-report (i.e., asking participants how they feel), or physiologically. Heart rate variability (HRV – i.e., the beat-to-beat variation in heart rate) is a well-evidenced physiological indicator of adaptive self- and ER (Appelhans and Luecken, 2006). According to polyvagal theory, during times of safety the parasympathetic nervous system dampens heart rate (HR) via the connection from the vagus nerve to the sino-atrial node in the heart, enabling proactive social activity (Porges, 2011). When threatened, the parasympathetic system retreats, allowing the sympathetic system to increase HR, and engage the orienting and defense system (Porges, 2007). The capacity of the vagus nerve to modulate HR in this manner is known as HRV: the more agile this system (and thus variable the HR), the more capacity the individual has to self-regulate, with a number of psychological and physiological benefits (Kemp and Quintana, 2013). If HRV is low, this indicates a system that is less adaptive with reduced regulation capacity. In experimental studies, measuring HRV is a useful indicator of the physiological benefit of engaging in various ER strategies (Appelhans and Luecken, 2006; Denson et al., 2011; Quintana et al., 2012; Geisler et al., 2013; Liddell and Courtney, 2018).

In the current study, participants in each cultural group were randomly assigned to be primed with a stress-induction scenario where the narrator engaged in either interpersonal or intrapersonal ER strategies. Following this, participants viewed high-arousing negative scenes in an emotion reactivity paradigm, while their HR was recorded and subjective ratings of emotional state were measured. In our second hypotheses, we predicted that the EA group would show greater benefit from implementing interpersonal ER strategies during the prime and while viewing negative cues compared to the WE group and relative to intrapersonal strategies. By contrast, the WE group would demonstrate greater benefit from implementing intrapersonal ER strategies during the task compared to the EA group and interpersonal strategies. This benefit would be reflected in (a) lowered subjective negative affect and (b) reduced HR and elevated HRV (indicating adaptive ER). In our third set of hypotheses, we predicted that individual differences in habitual use of interpersonal and interpersonal ER, and self-reported levels of individualistic or collectivistic self-construal, would moderate the degree that engagement in either interpersonal or intrapersonal ER strategies influenced negative affect or HR outcomes in each cultural group. For instance, habitual use of interpersonal ER strategies like perspective taking or social modeling might be expected to enhance the effect of the interpersonal prime.

## Materials and Methods

### Participants

One hundred and nineteen participants were recruited from the undergraduate psychology and international student pools at the University of New South Wales, Sydney, Australia. Participants were pre-screened for cultural background to ensure they had either WE (i.e., Caucasian) ancestry, or EA (i.e., Chinese, Japanese or Korean) ancestry. Further inclusion criteria included identity with a single cultural background (i.e., not bicultural) and for the EA group, living in Australia for less than 10 years. Fourteen participants had to be subsequently excluded due to incorrectly responding to pre-screening questions and not meeting cultural group inclusion criteria (of EA ancestry but living in Australia for more than 10 years (*n* = 6), participants who reported bicultural heritage (*n* = 4), and having South Asian (*n* = 1) or Southern/Eastern European (*n* = 3) ancestry). Participants were also subsequently excluded if they did not meet the inclusion criteria on a number of key factors that may affect HR and emotional responses which were measured during the study (see below): regular recreational or medicinal drug-use (*n* = 2); current smoker (*n* = 6); or scores on any of the subscales of the Depression Anxiety Stress Scale indicating extreme symptom severity (*n* = 8, DASS-21; Lovibond and Lovibond, 1995). A further three participants were excluded for incomplete self-report data or noisy ECG recordings.

Thus, a final sample of 86 participants completed the study (63 females, 23 males, *M* = 19.73 years old, *SD* = 3.55). There were 48 participants in the EA group (*n* = 23 randomly allocated to the interpersonal ER and *n* = 25 in the intrapersonal ER condition) and 38 participants in the WE group (*n* = 18 in the interpersonal ER and *n* = 20 in the intrapersonal ER condition). Even if excluded, participants received either course credit or AUD $15 in return for taking part in the study.

This study was carried out in accordance with the recommendations and approval of the University of New South Wales (UNSW) Human Research Ethics Advisory Panel C: Psychology. All participants gave informed consent in accordance with the Declaration of Helsinki.

### Measures

Participants were administered a number of self-report measures to index various aspects of culture and ER capacity.

The *Interpersonal Emotion Regulation Questionnaire* (IERQ) (Hofmann et al., 2016) measures how individuals engage with other people to regulate their emotions on four subscales: enhancing positive affect (e.g., *‘I like being around others when I’m excited to share my joy’*), perspective taking (e.g., *‘When I am annoyed, others can soothe me by telling me not to worry’*), soothing (e.g., *‘I look for other people to offer me compassion when I’m upset’*), and social modeling (e.g., *‘It makes me feel better to learn how others dealt with their emotions’*). Each item was rated on a 5-point Likert scale (1 = *not true for me at all*, to 5 = *extremely true for me*) and responses were summed within the four subscales. The original psychometric validation of the IERQ was conducted on a mixed cultural group cohort, including respondents with Asian ethnicity (Hofmann et al., 2016). The internal consistency for this sample was sound for all four subscales: enhancing positive affect (Cronbach α = 0.80), perspective taking (α = 0.73), soothing (α = 0.88), and social modeling (α = 0.87).

The *Emotion Regulation Questionnaire (ERQ)* is a 10-item measure of the use of intrapersonal ER strategies of reappraisal and emotional suppression to manage emotions in day-to-day life (Gross and John, 2003). Ratings were provided on a 7-point Likert scale, with responses reflecting typical experience and expression of emotions (1 = *strongly disagree*, to 7 = *strongly agree*). Adaptation of the ERQ to Asian youth populations indicate support for the original factor structure of the instrument (Liu et al., 2017). Responses are summed within the two sub-scales to provide reappraisal (Cronbach α = 0.71), and suppression scores (α = 0.74).

We used the extended 30-item version of the *Self-Construal Scale (SCS*; Singelis, 1994; Kitayama et al., 2014) to index individualistic and collectivistic self-construal. Responses were provided on a 7-point Likert scale (1 = *strongly disagree*, to 7 = *strongly agree*), with items summed within subscales. The internal consistency for this sample was sound for both subscales; collectivistic/interdependent (Cronbach α = 0.72) and individualistic/independent (α = 0.71). The SCS has been widely used and validated in EA populations (Singelis, 1994; Kitayama et al., 2014).

The following instruments were also administered in order to control for factors known to influence emotional reactivity to negative cues, ability to engage in imaginal exposure tasks and HR/HRV recordings, in analyses.

The *Visual Vividness Imagery Questionnaire* (Marks, 1973) was used to check for group differences in capacity to visualize images, and therefore engage in the prime task. Participants were asked to imagine four scenes (a country landscape, a friend or relative, a shop they have visited, and the sun rising), and were asked to rate how clearly they could visualize various visual components of these scenes on a 4-point Likert scale (1 = *not clearly at all*, to 4 = *can clearly imagine*). The VVIQ has been used in studies with EA participants (e.g., Nouchi, 2011). All items are summed to provide a single score, and internal consistency was high for this sample (Cronbach α = 0.88).

The DASS-21 is the short-form version of a self-report measure of symptoms of psychological distress within three subscales – depression, anxiety, and stress (Lovibond and Lovibond, 1995). Each symptom item was rated on the extent it applied to the previous week on a 4-point scale (0 = *did not apply to me at all*, to 3 = *applied to me very much, or most of the time*). Researchers have demonstrated cross-cultural construct validity of the DASS-21 in Asian groups (Norton, 2007). Participants’ responses to the items were summed for the three subscales to provide depression, anxiety, and stress scores.

Participants were asked to complete a number of measures indexing lifestyle factors that could influence cardiovascular activity (Reardon and Malik, 1996; Quintana et al., 2013a,b; Laborde et al., 2017). These included the Alcohol Use Disorders Identification Test (AUDIT-C; Rumpf et al., 2012), the International Physical Activity Questionnaire (IPAQ; Craig et al., 2003), and measures relating to caffeine consumption, illicit drug use, medical conditions and current medications.

Subjective changes in affect across the study were measured on the Positive and Negative Affect Scale (PANAS; Watson et al., 1998). Participants were presented with 20 emotional states (10 negative/10 positive), and asked to rate how they felt in relation to that state in the present moment (1 = very slightly/not at all, to 5 = extremely). Responses were summed separately for the positive and negative emotions. The PANAS has been validated for use in Chinese cultural groups (Huang et al., 2003). The internal consistency for baseline positive (Cronbach α = 0.89) and negative (α = 0.88) PANAS subscales was good, with similar consistency scores evident for all time points in the study.

A post-manipulation measure consisting of eight items was used to index the impact of the prime. These questions described different interpersonal [e.g., “I thought about how others (e.g., a friend) would react to the image to feel less upset, distressed or worried”] and intrapersonal (e.g., “I thought about the image with a more objective perspective to help me feel more calm”) ER strategies. Participants rated how frequently they engaged in each strategy during the emotion reactivity phase on a 5-point Likert scale (1 = *never, to* 5 = *very frequently)*, and internal consistency was satisfactory for the interpersonal (Cronbach α = 0.76) and the intrapersonal subscales (Cronbach α = 0.61).

### Stimuli

#### Prime Stimuli

Scenarios were developed to prime the implementation of either interpersonal or intrapersonal ER strategies when encountering a stressful experience. Each scenario featured the same first-person narrator approaching the scene of a motor vehicle accident on foot, with the narrator describing what they saw, how they felt and the ER strategies they engaged in. The two prime scenarios were matched on: their length, level of detail, the number of times ER strategies were used, and the ultimate outcome of applying the ER strategies (i.e., “this made me feel calmer”). In the interpersonal ER prime, various interpersonal ER strategies were described that included social modeling [e.g., *“They (emergency workers) do not look too troubled about the situation which helps ease my worry*” and “*I notice a girl from one of my lectures standing in the crowd looking at the cars. She looks in my direction and flashes me a kind smile. This helps me to feel calmer”*], perspective-taking and soothing (e.g., *“I recalled a friend telling me that they were able to reassure themselves that sometimes lights and sirens can make accidents seem much worse than they actually are, which helped him calm down*”). In the intrapersonal ER prime, various intrapersonal ER strategies were described including reappraisal (e.g., *“Instead I try to reassure myself that maybe the driver isn’t too badly hurt and this helps me easy my worry”*) and distraction (e.g., “*I try to distract myself by thinking about how the accident will be cleared up and everything will be back to normal by the time I leave university later that day. This helps me feel calmer”*). Each prime was pre-recorded using the same narrator and played to the participant via headphones, and were 2 min in duration.

#### Emotional Cues

During the image exposure phase, participants viewed a series of 20 negatively valenced and high arousing images selected from the International Affective Picture System (IAPS; Lang et al., 2008). Images depicted disaster scenes, physical injury or biological threats, and were specifically selected so that no scene included more than one person that may interfere with the interpersonal processes being activated.

### Procedure

Upon arrival at the psychophysiological laboratory at the University of New South Wales (UNSW Sydney), participants provided written informed consent as approved by the UNSW Human Research Ethics Advisory Panel C: Psychology. Following this, participant completed the DASS-21 and were also screened for a history of exposure to motor vehicle accident trauma (no participants endorsed this). Those who scored in the extremely severe range on either of the depression, anxiety or stress subscales of the DASS-21 were excluded at this stage (*n* = 8). Included participants then completed demographic questions, questions about their level of physical activity, and coffee, drug and alcohol consumption. Participants then moved onto the testing component of the study, which consisted of four phases.

#### Baseline Phase

Participants’ baseline mood was established through the completion of the PANAS (Watson et al., 1998). Electrocardiogram (ECG) leads and pads were attached to the participant’s chest and neck in order to record HR and HRV via ADInstruments Powerlab equipment and LabChart software (v. 8). After a short habituation period to the ECG leads, baseline HR and HRV were measured for 5 min, with participants resting in a seated, eyes open position.

#### Prime Phase

Participants listened to the prime narration via headphones according to their randomly assigned interpersonal or intrapersonal prime condition. The instructions asked participants to close their eyes and to try to vividly imagine that they were the narrator of the story, while HR/HRV was recorded. After the prime narration ended, participants again completed the PANAS.

#### Emotion Reactivity Phase

Participants were provided written instructions that asked them to think about the strategies the narrator used in the prime and to employ similar strategies when viewing the negative images to manage their own emotional reactions. Participants were then shown a series of highly arousing negative images for 5 min, while HR/HRV was recorded. A total of 20 images were randomly presented for 12 s each to allow enough time to process the scene and engage in ER strategies, followed by an inter-stimulus interval of three seconds where participants viewed a centralized fixation cross. Participants completed the PANAS after all the images were presented.

#### Recovery Phase

Immediately following the exposure phase, participants entered the recovery-resting phase while HR/HRV was recorded for 5 min. Participants completed a series of post-manipulation questions and the remaining self-report measures including the IERQ, ERQ, SCS, and VVIQ, described above. Following this, participants were fully debriefed.

### Data Analysis

#### Data Cleaning and Pre-processing

ECG data was processed in Labchart (v. 8). R-peaks of the QRS complex were automatically detected to compute HR (R-peaks per minute) and HRV variables (based on the time interval variation between R-peaks). R-peak markers were manually checked and corrected where necessary (e.g., removal of movement artifacts, addition of missed beats). High frequency power (absolute and normalized units) was the primary HRV variable of interest (HF-HRV), as it is a central indicator of parasympathetic modulation of the heart (Bernston et al., 1997; Appelhans and Luecken, 2006) and reflects adaptive ER (Appelhans and Luecken, 2006; Geisler et al., 2010; Knepp et al., 2015).

Skewness and kurtosis measures for HF-HRV (absolute) indicated an abnormal distribution, and thus, HF-HRV (absolute) data were natural log-transformed (note, this was not necessary for HF-HRV normalized units). All data [HR, HF-HRV (abs/norm)] were then screened for outliers (defined as more than three standard deviations from the sample mean). Outliers were then replaced by either the maximum (three standard deviations above the mean) or minimum integer (three standard deviations below the mean; comprising 1.89% of the total transformed data). This method is commonly used in psychophysiological studies to prevent outliers from unduly influencing statistical outcomes but also to retain the overall distribution in the data (Howell, 1998; Laborde et al., 2017; Liddell and Courtney, 2018). Next, difference scores were calculated for HF-HRV and HR comparing each of the active phases to the baseline phase (prime phase = priming phase – baseline; emotion reactivity phase = image exposure phase – baseline; and recovery phase = recovery phase – baseline). This data analysis method is commonly applied in HRV studies (Laborde et al., 2017). Outliers were again checked in the difference score data (again, defined as more than three standard deviations from the sample mean), and none were detected.

#### Preprocessing PANAS Data

Difference scores comparative to baseline for the three active phases were also calculated for positive and negative affect, similar to the HR/HRV data. Levene’s test of equality of error variance was statistically significant for positive affect during the prime phase. Therefore, the data was transformed through replacing outliers according to a strict protocol (1.5 standard deviations below or above the mean), with a total of 6.9% of the positive prime difference scores being replaced.

### Statistical Analysis

Data was analyzed using SPSS (v. 24). Firstly, independent *t*-tests were carried out to verify that the cultural groups differed on the subscales of the IERQ, ERQ, and SCS to reflect previous theoretical and empirical findings. Then cultural group (WE vs. EA) and manipulation group (interpersonal vs. intrapersonal) differences on self-report measures of interpersonal and intrapersonal ER, self-construal, demographics and habits (e.g., alcohol consumption) that impact HR were examined using one-way ANOVAs comparing the four experimental groups for continuous data (alpha level of *p* < 0.05) and chi-square tests for categorical data (*p* < 0.05). For continuous measures, *post hoc* contrasts tested whether significant differences detected were between cultural or ER prime groups (Bonferroni-corrected).

Next, a mixed-model 2 × 2 × (3) MANCOVA was conducted to examine between and within-group effects on HR, HRV and self-reported affect difference scores (i.e., each phase compared to baseline described above) as a function of culture and ER prime groups (alpha level of *p* < 0.05). Age, alcohol and anxiety scores were included as covariates as these three factors were found to be significantly different between groups, and are known to effect HR and HRV (Reardon and Malik, 1996; Quintana et al., 2013a,b; Chalmers et al., 2014).

*Post hoc* 2 (WE vs. EA) × 2 (interpersonal vs. intrapersonal) ANCOVAs were subsequently conducted to examine between-group effects at each phase (prime, image, and recovery) separately, followed by pairwise comparisons to determine the direction of the significant effects (*p*-values were Bonferroni-corrected).

Finally, a series of regression analyses were conducted to determine the relative contribution of individual differences in self-construal (collectivistic, individualistic self-construal) and habitual self-reported use of interpersonal (perspective taking, social modeling, soothing, enhancing positive affect) and intrapersonal (reappraisal, suppression) ER strategies on HF-HRV during each phase of the study. As such, hierarchical moderated regression analyses were conducted for the two cultural groups separately. Predictors were mean centered to correct for multicollinearity. Two-way interaction terms were included in the model to examine the interaction between prime group and each predictor. Initial hierarchical moderated regression models consisted of three steps: (1) prime group; (2) independent mean centered predictors; (3) interaction terms between prime group and each predictor value. From these initial regression models, significant predictors were identified and included in final regression models. A significant predictor in the initial model was defined as any predictor with a *p*-value less than 0.1; in the final models, significant predictors were determined at *p* < 0.05. Simple slopes analyses were carried out as *post hoc* tests to further examine the meaning of a significant interaction term (adjusted *p* < 0.025, corrected for testing two slopes per interaction based on the two manipulation groups).

## Results

### Participant Characteristics

Table 1 presents the demographic and self-report measures for the four groups (cultural group by prime group). No significant group differences were observed in terms of gender, physical activity or visual imagery capacity. The EA group had been resident in Australia for an average of 2.40 years (*SD* = 2.78). Chi-square tests indicated that were no significant group differences between caffeine [χ^2^ (9) = 8.42, *p* = 0.49] or energy drink [χ^2^ (6) = 5.48, *p* = 0.48] consumption. Groups significantly differed in terms of age, with *post hoc* contrasts indicating that those randomized to the interpersonal prime condition were older than those in the intrapersonal prime condition [*t*(59.9) = 2.20, *p* = 0.023]. Groups were similar on the depression and stress subscales of the DASS, but differed in terms of anxiety, with the EA group reporting higher anxiety scores than the WE group [*t*(67.01) = −3.10, *p* = 0.003]. Groups also differed in terms of alcohol consumption, with *post hoc* contrasts demonstrating that the WE group reported higher levels of alcohol use than the EA group [*t*(82) = 6.89, *p* < 0.001]. As such, subsequent ANOVA tests controlled for group differences in age, anxiety and alcohol use.

**Table 1:** Group demographics and characteristics.

	Western European group (*n* = 38)		East Asian group (*n* = 48)		
	Interpersonal prime (*n* = 18) *M* (*SD*)	Intrapersonal prime (*n* = 20) *M* (*SD*)	Interpersonal prime (*n* = 23) *M* (*SD*)	Intrapersonal prime (*n* = 25) *M* (*SD*)	*F* (*p*)
**Age**	19.39 (1.58)	19.05 (1.54)	21.52 (4.11)	18.88 (4.61)	2.91 (0.04)^∗^
**Alcohol**	4.44 (2.31)	4.05 (1.85)	1.43 (1.78)	1.36 (1.73)	15.09 (0.001)^∗^
**Interpersonal ER (IERQ)**					
Enhancing positive affect	19.89 (3.07)	19.20 (3.27)	17.26 (3.34)	16.36 (3.59)	5.14 (0.003)^∗^
Perspective tasking	11.00 (4.13)	11.85 (5.08)	13.61 (3.17)	14.84 (2.76)	4.42 (0.001)^∗^
Soothing	15.11 (4.60)	14.05 (6.45)	14.08 (5.09)	14.88 (3.62)	0.25 (0.87)
Social modeling	14.17 (4.57)	13.35 (5.61)	17.04 (4.19)	16.04 (4.15)	2.85 (0.04)^∗^
**Intrapersonal ER (ERQ)**					
Reappraisal	33.28 (4.21)	30.95 (4.21)	31.09 (5.72)	32.36 (4.68)	1.06 (0.37)
Suppression	13.56 (5.80)	16.40 (4.25)	16.70 (3.90)	17.20 (4.02)	2.62 (0.10)
**Self-construal (SCS)**					
Individualism	68.7∖8 (9.99)	71.10 (8.87)	73.70 (9.47)	68.68 (7.69)	1.57 (0.20)
Collectivism	71.39 (9.75)	68.20 (9.13)	74.78 (8.11)	72.76 (7.64)	2.21 (0.10)
**Physical activity score**	2109.00 (1933.87)	2944.97 (2976.88)	2555.09 (2910.29)	2370.56 (1989.82)	0.38 (0.77)
**DASS-21**					
Depression	1.78 (2.31)	2.90 (2.73)	2.35 (2.12)	2.40 (2.63)	0.70 (0.55)
Anxiety	1.28 (1.13)	2.30 (2.81)	3.35 (2.60)	3.32 (2.41)	3.49 (0.02)^∗^
Stress	4.22 (3.98)	4.80 (4.62)	4.30 (3.14)	3.64 (2.66)	0.39 (0.76)
**Imagery score (VVIQ)**	8.58 (1.65)	8.84 (2.67)	9.62 (2.74)	9.64 (2.30)	1.05 (0.37)
**Post-manipulation questions**					
Interpersonal score	1.94 (0.73)	1.61 (0.67)	2.46 (0.83)	1.91 (0.67)	4.92 (0.003)^∗^
Intrapersonal score	3.46 (0.79)	3.70 (0.72)	3.23 (0.92)	2.99 (0.87)	2.48 (0.07)
Suppression score	3.14 (1.06)	3.48 (1.24)	3.39 (1.03)	3.16 (1.11)	0.37 (0.77)
Reappraisal/Distraction score	3.10 (0.94)	3.17 (1.11)	3.57 (1.24)	3.36 (1.08)	0.51 (0.68)
**Gender distribution**	***n***	***n***	***n***	***n***	**χ^2^*(p)***
Males	4	2	8	9	4.90 (0.18)
Females	14	18	15	16	

*One-way ANOVA findings presented in right column; ^∗^p < 0.05.*

In regards to self-construal, the one-way ANOVA did not reveal significant differences, but we did not expect there to be differences between prime group. Considering just cultural group, *t*-tests revealed that the EA group reported higher levels of collectivistic self-construal compared to the WE group [*t*(84) = −2.16, *p* = 0.03], but no group difference in individualistic self-construal was observed [*t*(84) = 0.55, *p* = 0.58].

### Manipulation Checks

Participants answered post-prime manipulation questions to indicate whether the experimental prime had an influence on the ER strategies used during the experiment. A one-way ANOVA conducted across the four groups indicated that there was a difference in the self-reported use of interpersonal ER strategies between groups [*F*(3,82) = 4.92, *p* = 0.003]. *Post hoc* contrasts indicated a significant main effect of cultural group: EA participants reported using interpersonal strategies more frequently during the experiment than WE participants, regardless of their manipulation group [*t*(82) = −2.61, *p* = 0.011]. There was also a significant main effect of manipulation condition. Participants in the interpersonal condition reported using interpersonal strategies more than participants in the intrapersonal group [*t*(82) = 2.50, *p* = 0.015], suggesting the prime narration activated interpersonal ER strategies. However, a similar effect was not observed for those in the intrapersonal group (suppression – *t*(82) = −1.06, *p* = 0.29; reappraisal/distraction – *t*(82) = 0.46, *p* = 0.65).

### Hypothesis 1: Group Differences in Habitual Use of Interpersonal and Intrapersonal ER Strategies

There were no significant differences between the four groups in habitual use of intrapersonal ER strategies (Table 1), and cultural main-effect *t*-tests also did not reveal any significant differences in self-reported use of reappraisal [*t*(84) = 0.29, *p* = 0.77] or suppression [*t*(84) = 1.94, *p* = 0.056] between groups. Notably, there was a marginally significant effect for the suppression condition, indicating a trend toward the EA group reporting greater use of suppression relative to the WE group.

The ANOVA revealed that there were statistically significant group differences on three of the IERQ subscales: *enhancing positive affect*, *social modeling*, and *perspective taking* (*p* < 0.05). *Post hoc* contrast tests showed EA participants had higher *perspective taking* [*t*(58.06) = −3.24, *p* < 0.001] and *social modeling* scores [*t*(82) = −2.77, *p* = 0.01] than WE participants, averaged across manipulation groups, suggesting that EA group more commonly engaged in these two core interpersonal ER strategies in daily life. *Post hoc* contrast tests also revealed that WE participants had higher *enhancing positive affect* scores than EA participants [*t*(82) = 3.76, *p* < 0.001].

### Hypothesis 2a: Cultural and Prime Group Differences in Self-Reported Affect

To determine whether groups differentially benefited from engaging in interpersonal vs intrapersonal ER following the prime, a 2 × 2 × (3) MANCOVA was conducted. The between-subjects factors were cultural group (WE and EA), and prime group (interpersonal and intrapersonal), and the within-subjects factor was experimental phase (prime, emotion reactivity and recovery). Covariates were age, alcohol and anxiety scores. The dependent variables were self-reported positive and negative affect change (relative to baseline).

A significant cultural group by phase interaction was detected for positive affect [*F*(2,79) = 6.07, *p* = 0.003]. *Post hoc* MANCOVAs conducted within each phase found that while cultural group differences were not detected during the prime phase [*F*(1,79) = 3.58, *p* = 0.062], significant main effects of culture on positive affect change following the emotion reactivity [*F*(1,79) = 6.76, *p* = 0.011] and recovery phases (F(1,79) = 10.25, p=.002) were evident (Bonferroni-corrected). This effect was due to stronger decreases in positive affect being reported in the EA group compared to the WE group during emotion reactivity and recovery relative to baseline. No significant between-group or interaction effects were observed for the negative affect change scores.

### Hypotheses 2b: Cultural and Prime Group Differences in HR/HRV Changes During Prime, Emotion Reactivity, and Recovery Phases

The same 2 × 2 × (3) MANCOVA analysis as above, but with HRV and HR as the dependent variables, was conducted. Covariates were age, alcohol, and anxiety scores.

No significant three-way or two-way interaction effects for either measure were observed. Significant between-subjects main effects of cultural group on HF-HRV [absolute – *F*(3,82) = 9.39, *p* = 0.003; normalized – *F*(3,82) = 5.06, *p* = 0.02] and HR [*F*(3,82) = 16.69, *p* < 0.001] were found, indicating that the EA group demonstrated elevated HR-HRV and reduced HR relative to the WE group across all phases of the study. There was also a significant main effect of prime group on HR [*F*(3,82) = 4.77, *p* = 0.03], such that the intrapersonal prime group showed greater decreases in HR relative to baseline across all phases of the study compared to the interpersonal prime group.

Since there were no significant within-subjects effect of Phase, three *post hoc* 2 (Cultural group: WE vs. EA) × 2 (Manipulation group: Interpersonal vs. Intrapersonal) ANCOVAs were conducted for each phase separately, with a Bonferroni corrected threshold of *p* < 0.0167. Follow-up pairwise comparisons were used to test the direction of any significant interaction effects (*p* < 0.05, Bonferroni-corrected).

#### Prime Phase

**FIGURE 1:**
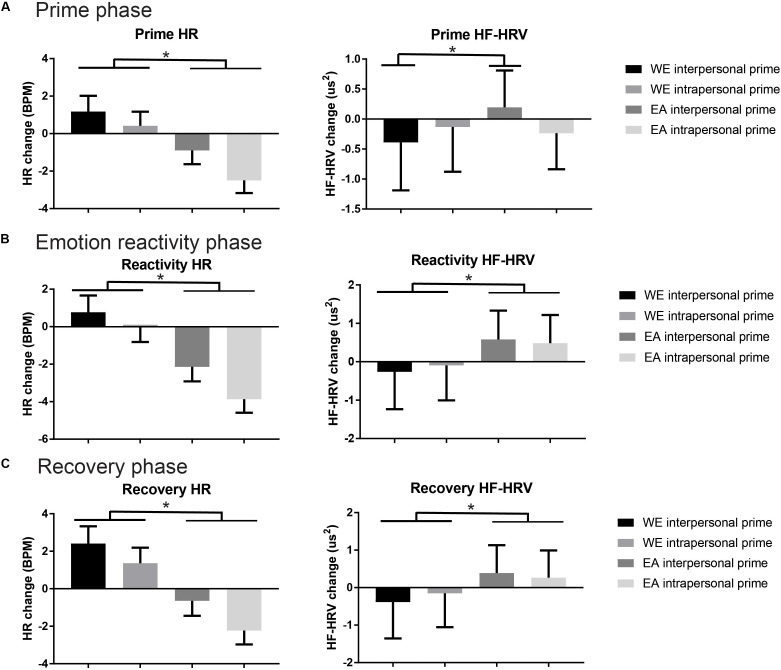
Heart rate (HR) and high-frequency power heart rate variability (HF-HRV, absolute units) mean values across the three phases of the experiment: **(A)** prime phase; **(B)** emotion reactivity phase; **(C)** recovery phase. Data is presented for cultural group by prime group, with significant main or interaction effects marked by ^∗^(*p* < 0.05 Bonferroni corrected).

A significant between-subjects cultural and prime group interaction for HF-HRV during the prime phase was observed [significant in absolute units only – *F*(1,85) = 6.22, *p* = 0.015; normalized units – *F*(1,85) = 1.67, *p* = 0.20]. Follow-up pairwise comparisons indicated that EA participants showed elevated HF-HRV (absolute units) compared to WE participants in the interpersonal ER condition (*p* = 0.018). A significant between-subjects cultural group main effect at the prime phase on HR [*F*(1,85) = 7.46, *p* = 0.01], revealed greater decreases in HR relative to baseline in the intrapersonal ER condition across groups; see Figure 1A.

#### Emotion Reactivity Phase

Significant cultural group main effects during the emotion reactivity phase were observed for HR [*F*(1,85) = 12.20, *p* = 0.001] and HF-HRV [absolute – *F*(1,85) = 10.33, *p* = 0.002, and normalized units – *F*(1,85) = 7.46, *p* = 0.01]; see Figure 1B. EA participants showed a relative reduction in HR, and increase in HF-HRV compared to the WE group, regardless of prime manipulation. No significant interaction effects were observed.

#### Recovery Phase

A cultural group main effect at the recovery phase on HR [*F*(1,85) = 10.99, *p* = 0.001] and HF-HRV [absolute *F*(1,85) = 7.39, *p* = 0.01] was detected. Again, EA participants showed reduced HR and elevated HF-HRV compared to WE participants, but no interaction effects with prime group were found (Figure 1C).

### Hypotheses 3: The Effect of Individual Differences in Self-Reported ER and Self-Construal on HF-HRV in Western European and East Asian Groups

Moderated hierarchical regression models were constructed to examine the effect of prime group, self-reported self-construal, and habitual use of interpersonal and intrapersonal ER strategies on HF-HRV (absolute) changes during the prime, emotional reactivity and recovery phases of the study. We focused on HF-HRV (absolute units) as the key dependent variable as this was where we observed a cultural by prime group interaction in the between-group ANCOVA analysis (prime phase only).

#### East Asian Group

Table 2 presents the final models for each phase.

**Table 2:** Final moderated regression models for the East Asian group across the prime, emotion reactivity, and recovery phases of the study; ^∗^*p* < 0.05.

Model	Unstandardized coefficients		Standardized coefficients		
	*B*	*SE*	β	*t*	Significance
**Predicting HF-HRV during prime phase**					
*Step 1*					
Constant	−0.187	0.105		−1.779	0.082
Prime group	0.410	0.151	0.370	2.705	0.010^∗^
*Step 2*					
Constant	−0.185	0.109		−1.697	0.097
Prime group	0.426	0.161	0.386	2.644	0.011^∗^
Collectivistic self-construal	−0.002	0.011	−0.027	−0.173	0.863
Interpersonal ER: Soothing	−0.011	0.023	−0.087	−0.489	0.627
Interpersonal ER: Social modeling	0.012	0.025	0.087	0.463	0.646
*Step 3*					
Constant	−0.174	0.104		−1.673	0.102
Prime group	0.377	0.161	0.341	2.336	0.024^∗^
Collectivistic self-construal	−0.025	0.015	−0.353	−1.631	0.111
Interpersonal ER: Soothing	0.001	0.022	0.011	0.063	0.950
Interpersonal ER: Social modeling	0.054	0.030	0.399	1.800	0.079
Prime group × social modeling Interaction	−0.097	0.041	−0.518	−2.377	0.022^∗^
Prime group × collectivistic self-construal interaction	0.043	0.021	0.441	2.012	0.051
**Predicting HF-HRV during emotion reactivity phase**					
*Step 1*					
Constant	0.380	0.152		2.501	0.016^∗^
Prime group	0.131	0.219	0.088	0.596	0.554
*Step 2*					
Constant	0.354	0.151		2.290	0.027^∗^
Prime group	0.234	0.225	0.157	1.039	0.304
Individualistic self-construal	0.021	0.013	0.242	1.603	0.116
**Step 3**					
Constant	0.272	0.139		1.952	0.057
Prime group	0.201	0.205	0.135	0.981	0.332
Individualistic self-construal	0.065	0.018	0.762	3.591	0.001^∗^
Prime group × individualistic self-construal	−0.076	0.024	−0.676	−3.215	0.002^∗^
**Predicting HF-HRV during recovery phase**					
*Step 1*					
Constant	0.193	0.158		1.226	0.227
Prime group	0.110	0.228	0.071	0.482	0.632
*Step 2*					
Constant	0.186	0.161		1.154	0.255
Prime group	0.133	0.240	0.086	0.553	0.583
Individualistic self-construal	0.005	0.014	0.052	0.335	0.739
*Step 3*					
Constant	0.146	0.161		0.906	0.370
Manipulation group	0.115	0.237	0.074	0.486	0.629
Individualistic self-construal	0.29	0.021	0.324	1.370	0.178
Prime group × individualistic self-construal	−0.041	0.027	−0.353	−1.507	0.139

##### Prime phase

The final model was significant [*F*(2,41) = 2.476, *p* = 0.039, Adj *R*^2^ = 0.16] and accounted for 26.6% of the variance. The prime group was a significant predictor, indicating that EA in the interpersonal prime condition showed elevated HF-HRV compared to those in the intrapersonal condition.

**FIGURE 2:**
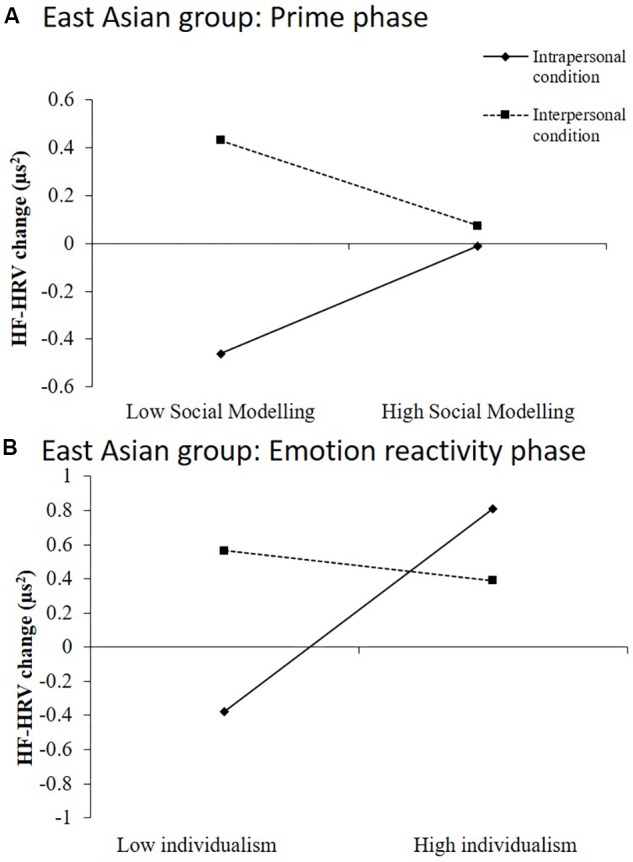
Significant interaction effects resulting from hierarchical moderated regression analyses performed with data from the East Asian group. **(A)** Habitual use of social modeling interacted with ER prime condition in the East Asian group. **(B)** Trait level of individualistic self-construal interacted with ER prime condition in the East Asian group.

A significant prime group by social modeling interpersonal ER strategy was also observed. Simple slopes analysis showed that in the interpersonal condition, there was no difference between low or high social modeling (*B* = −0.04, *t* = −1.36, *p* = 0.18). However, in the intrapersonal prime group, a trend effect was observed where those with low social modeling showed a greater decrease in HF-HRV power relative to baseline during the prime compared to those with high social modeling skills (*B* = 0.05, *t* = 1.71, *p* = 0.09); see Figure 2A.

##### Emotion reactivity phase

The final model was significant at the third step [*F*(3,44) = 4.63, *p* = 0.01, Adj *R*^2^ = 0.19] and accounted for 24% of the variance of change in HF-HRV during emotion-reactivity in the EA group. Individualistic self-construal in interaction with prime group emerged as the strongest predictor. Simple slopes analysis showed that levels of individualism did not affect HF-HRV in the interpersonal prime condition (*B* = −0.01, *t* = −0.17, *p* = 0.86), however, higher individualism predicted greater change in HF-HRV relative to baseline for those in the intrapersonal prime group (*B* = 0.06, *t* = 2.06, *p* = 0.046; not significant at corrected level); see Figure 2B.

##### Recovery phase

The final model was not significant (*p* > 0.05), and there were no significant predictor variables.

#### Western European Group

Table 3 presents the final models for each phase for the WE group.

**Table 3:** Final moderated regression models for the Western European group across prime, emotion reactivity, and recovery phases of the study; ^∗^*p* < 0.05.

Model	Unstandardized coefficients		Standardized coefficients		
	*B*	*SE*	β	*t*	Significance
**Predicting HF-HRV during prime phase**					
*Step 1*					
Constant	0.216	0.123		−1.759	0.087
Interpersonal ER: Perspective taking	−0.053	0.025	−0.330	−2.100	0.043^∗^
**Predicting HF-HRV during emotion reactivity phase**					
*Step 1*					
Constant	−0.003	0.167		−0.016	0.987
Prime group	−0.123	0.243	−0.084	−0.509	0.614
*Step 2*					
Constant	−0.075	0.175		−0.428	0.671
Prime group	−0.010	0.259	−0.007	−0.039	0.970
Collectivistic self-construal	0.012	0.014	0.158	0.868	0.392
Intrapersonal ER: Reappraisal	0.046	0.030	0.266	1.528	0.136
Interpersonal ER: Perspective taking	0.005	0.034	0.029	0.135	0.894
Interpersonal ER: Soothing	−0.026	0.028	−0.200	−0.956	0.346
*Step 3*					
Constant	−0.129	0.178		−0.725	0.474
Prime group	0.112	0.284	0.077	0.395	0.695
Collectivistic self-construal	0.018	0.015	0.233	1.218	0.233
Intrapersonal ER: Reappraisal	0.023	0.033	0.133	0.680	0.502
Interpersonal ER: Perspective taking	0.055	0.047	0.343	1.171	0.251
Interpersonal ER: Soothing	−0.058	0.037	−0.440	−1.573	0.127
Prime group × soothing	0.050	0.057	0.214	0.890	0.381
Prime group × perspective taking	−0.109	0.069	−0.444	−1.583	0.124
Prime group × collectivistic self-construal	0.019	0.020	0.175	0.966	0.342
**Predicting HF-HRV during recovery phase**					
*Step 1*					
Constant	−0.071	0.106		−0.666	0.510
Intrapersonal ER: Reappraisal	0.051	0.024	0.325	2.157	0.038^∗^
Interpersonal ER: Perspective taking	−0.044	0.022	−0.299	−1.988	0.055

##### Prime phase

The final model was significant [*F*(1,36) = 4.41, *p* = 0.043, Adj *R*^2^ = 0.08] and accounted for 10.9% of the variance in HF-HRV change during the prime phase for the WE group. Perspective taking was the only significant predictor as a main effect, suggesting that the lower the self-reported perspective-taking, the greater the change in HF-HRV during priming across both conditions.

##### Emotion reactivity phase

The final model was not significant (*p* > 0.05), and there were no significant predictor variables.

##### Recovery phase

The final model was significant [*F*(2,35) = 4.96, *p* = 0.01, Adj *R*^2^ = 0.18] and accounted for 22.1% of the variance in HF-HRV change during recovery. Higher levels of trait reappraisal and lower levels of perspective taking were associated with elevated HF-HRV relative to baseline during the recovery phase in the WE group.

## Discussion

This study examined whether culture moderated the benefit of engaging interpersonal ER strategies during a prime and emotion reactivity task. The findings support the notion that an EA cultural group with stronger collectivistic self-construal, reported greater habitual engagement in social modelling and perspective taking interpersonal ER strategies compared to a WE group. Moreover, priming interpersonal ER strategies to manage emotional reactions to negative stimuli increased HF-HRV – an indicator of parasympathetic innervation of the heart and of stronger ER – in the EA compared to the WE group during exposure to the prime itself, but not during the subsequent emotion reactivity or recovery phases. This suggests that the EA group benefited more from interpersonal priming relative to the WE group in terms of physiological responding, but that this did not extend beyond the immediate prime exposure phase. Furthermore, the EA group demonstrated generally higher HF-HRV and lowered HR during reactivity and recovery phases compared to the WE group, indicating the EA group was better at implementing both interpersonal and intrapersonal ER strategies to moderate physiological responses to negative cues. The findings also support the role of individual differences in self-construal, interpersonal and intrapersonal ER styles in modulating HF-HRV responses across the phases of the experiment within each cultural group.

We observed that EA participants reported relatively higher scores on the IERQ subscales of social modeling (i.e., understanding how others handled situations to assist in one’s own ER) and perspective taking (i.e., having others put emotional situations into perspective in order to self-regulate strong emotions; Hofmann et al., 2016), compared to the WE group. This finding is consistent with the notion that cultural groups high in collectivistic self-construal, and that place strong personal value on interpersonal connectivity and group harmony, benefit from *implicit* forms of social support (such as calling to mind a supportive person) when managing stress reactions (Taylor et al., 2007). EA cultural groups also appear to more readily take other’s perspective in social or even self-reflective situations (Sul et al., 2012; Ma et al., 2014), suggesting engaging in interpersonal ER strategies may be more socioculturally adaptive for this group. By contrast, we observed that WE reported higher scores on the enhancing positive affect sub-scale of the IERQ (i.e., measuring an individual’s tendency to seek out others to increase their own positive emotional states) (Hofmann et al., 2016). This finding reflects cultural research demonstrating that WE individuals are motivated to increase positive affect but EA groups are driven by decreasing negative affect when regulating emotion (Lee et al., 2000). It also accords with previous findings that WE cultural groups prefer to express positive affect through high arousal emotions such as excitement, as opposed to EA group preferences toward low arousal positive emotions such as calmness (Tsai et al., 2006; Matsumoto et al., 2008). Curiously, we did not observe strong cultural group differences in engagement in reappraisal or suppression intrapersonal ER strategies, only observing a trend toward elevated suppression in the EA group – consistent with previous findings (Ford and Mauss, 2015). Overall, the self-report findings support the idea that ER strategies may be shaped by culture in order to ensure adaptive emotional expression, reactions and management (De Leersnyder et al., 2013).

The main experimental finding that emerged was that the EA group, when primed with interpersonal ER strategies, showed patterns of elevated HF-HRV and reduced HR during the prime phase, relative to the WE group. This pattern is indicative of adaptive ER, and are in partial support for our hypotheses. Previous research has shown that individuals benefit most from engaging in ER strategies that are congruent with their cultural background (Murata et al., 2013) or trait ER style (Nickerson et al., 2017). However, contrary to hypotheses, we did not observe that WE participants primed with intrapersonal ER strategies showed cardiovascular patterns reflecting adaptive ER. Nor did we find the cultural group difference observed in the prime phase was maintained across the emotion reactivity or recovery phases. These null findings may reflect the limitation of the prime methodology to evoke differential ER strategy engagement (as opposed to direct instructed ER; see limitations below for further discussion on this issue). Instead, we observed a general pattern whereby the EA group exhibited elevated HF-HRV and decreased HR during both reactivity and recovery phases compared to the WE group, irrespective of the prime condition. This finding suggests that the EA group was generally better at down-regulating their emotional reactions over the course of the study in both prime groups. This is consistent with previous findings demonstrating that EA participants experienced more adaptive cardiovascular patterns during an anger provocation task compared to WE participants (Mauss and Butler, 2010). The findings may reflect the idea that down-regulating emotional reactions are more consistent with EA collectivistic cultural expectations, whereas WEs are more motivated to express their emotions in line with their own well-being and thus, do not have the same tendencies to control their emotional expressions (Murata et al., 2013). Since we did not measure emotional control, this idea will need to be examined in future studies.

Another interpretation of the findings is that the EA participants were simply engaging in interpersonal ER strategies, regardless of their allocated prime condition – as indicated by the manipulation check results. If they did so, such a strategy may explain why the EA group demonstrated signs of more adaptive physiological and regulatory responses via increased HRV and reduced HR across all phases of the study, relative to baseline. Future studies could consider implementing a direct instruction paradigm, rather than suggested engagement via a prime task, to increase experimental control over the specific strategies used by participants.

In terms of self-reported affect across the experiments, no significant differences were found on negative affect between the groups. However, the WE group showed smaller decreases in self-reported positive affect in the emotion reactivity and recovery phases compared to the EA group. This finding accords with our other result that the WE group reported stronger habitual engagement in the enhancing positive affect interpersonal ER strategy. The finding that retaining positive affect was stronger in the WE group also reflects the notion that positive affect is a more desirable emotional state for WE groups compared to EA groups (Diener et al., 1995; Matsumoto and Hwang, 2012), and that this group is generally more motivated to increase positive affect via ER (Lee et al., 2000).

Individual differences in self-construal and habitual use of intrapersonal and interpersonal ER strategies modulated HF-HRV reactions across the time course of the experiment between groups. Specifically, for the EA group, lower levels of social modeling predicted greater decreases in HF-HRV relative to baseline during exposure to the intrapersonal prime compared to high social modeling, in a trend-level effect. This pattern was expected in that those with higher social modeling traits would benefit more from the interpersonal prime by exhibiting relatively higher HF-HRV during the prime and emotion reactivity phases. This finding indicates that trait interpersonal ER skills may protect against the cardiovascular cost of threat exposure when interpersonal ER skills are engaged. However, further research will be required to investigate this notion further.

During the emotion reactivity phase, it was also observed that those in the EA group with higher individualistic self-construal showed increased HF-HRV reflecting adaptive ER in the intrapersonal prime condition. This suggests that there are some in the EA group where intrapersonal strategies are beneficial to coping with exposure to negative emotional cues, particularly if those strategies are consistent with their individualistic self-construal (De Leersnyder et al., 2014; Ford and Mauss, 2015). This finding highlights that while the EA group may preference interpersonal ER strategies, there may be individual differences that modulate the effect of alternative ER approaches. We note that interactions between the prime condition and both social modeling and individualism are difficult to directly compare, given that social modeling was manipulated by the prime, whereas individualistic self-construal was not, and that the interactions were observed during different phases of the experiment.

Amongst the WE group, only main effects were observed as individual differences did not interact with prime group. Higher levels of perspective taking predicted decreased HF-HRV (i.e., less adaptive ER) during both priming and recovery phases in the WE group. Previous studies have found the use of ER strategies associated with consideration of others (i.e., suppression) or placing high value on interpersonal harmony was negatively correlated with the wellbeing of WE individuals (Wei et al., 2013). Similarly, the association between high perspective taking and decreases in HRV in the current study could be due to the fact that perspective taking is culturally incongruent for WE individuals whose culture places value on unique personal experiences (De Leersnyder et al., 2013). Additionally, high trait reappraisal predicted increased HF-HRV during the recovery phase. Past research has highlighted that people with WE cultural backgrounds have a greater tendency to engage in and benefit from cognitive reappraisal (Gross, 2013; Ford and Mauss, 2015). Theoretically, this has been linked to the value placed on individual emotional experiences and autonomous expression (Matsumoto et al., 2008). Therefore, the relationship between trait reappraisal and elevated HRV in the WE group further demonstrates the benefit of culturally congruent ER processes.

The findings of this study must be interpreted within the context of a number of limitations. We found that the prime condition did not modulate cardiovascular or affect indices beyond the prime exposure phase, suggesting that the priming method employed in this study may not be the most effective approach for studying the effect of engaging in interpersonal ER strategies to manage emotional reactions to negative cues. This approach was selected to maximize the study of implicit cultural differences in ER strategy engagement, due to previous research demonstrating the maladaptive effects of explicitly instructing ER strategies if these strategies are incongruent to trait ER style (Nickerson et al., 2017). Future studies could consider implementing a direct instructed ER condition to enhance interpersonal ER engagement. A related methodological limitation was the fact that a different modality was implemented for the prime compared to the emotion reactivity phase. The interpersonal prime describes how the narrator used other people as reference points to help them feel calmer about the car accident scenario. However, during the image exposure task, participants did not have a similar reference of how others would react to the images to help them manage their emotional response, and thus, were forced to rely on their own internal reference systems. This may explain why the prime conditions did not continue to affect cardiovascular or affective responses during the emotion reactivity phase. The use of new technology such as virtual reality could be one way in which to optimize the ecological validity of engaging in interpersonal ER strategies in future studies. Another limitation was the short recording period of the prime phase, which was for 2 min, compared to the 5-min recording time of baseline, reactivity and recovery phases. While it has been recommended that HRV recordings be at least 5 min (Bernston et al., 1997), recent studies have found strong correlations between HF-HRV extracted from ultra-short recording periods (i.e., 1 min) and those from longer recording periods (i.e., 5 min) (Schroeder et al., 2004; Nussinovitch et al., 2011, 2012). One 1 min records are now recommended as minimum for HF-HRV indices (Shaffer and Ginsberg, 2017).

## Conclusion

To our knowledge, this is the first study to consider cultural differences in interpersonal ER. The findings support the notion that an EA cultural group reported stronger engagement in interpersonal ER strategies such as social modeling, and demonstrated cardiovascular indicators of more adaptive ER responses during an interpersonal ER prime, compared to a WE group. The findings highlight the potential for interpersonal ER strategies to enhance the management of emotional reactions particularly in collectivistic cultural groups, adding to the literature that the benefit of various ER strategies may be dependent on various contextual factors – including cultural congruence.

## Ethics Statement

The research was approved by the UNSW Human Research Ethics Advisory Panel C: Psychology. Participants provided written informed consent after reading the Participant Information and Consent Form, and had the freedom to withdraw from the study at any time. Once completed, participants were fully debriefed and received either course credit or AUD $15 reimbursement.

## Author Contributions

BL conceptualized and designed the study, supervised data collection and analysis, and wrote the manuscript. EW conceptualized and designed the study, conducted data collection and analysis, and contributed to the manuscript based on her Honors in Psychology thesis.

## Conflict of Interest Statement

The authors declare that the research was conducted in the absence of any commercial or financial relationships that could be construed as a potential conflict of interest.
